# Simulation Analysis of a Wavefront Reconstruction of a Large Aperture Laser Beam

**DOI:** 10.3390/s23020623

**Published:** 2023-01-05

**Authors:** Gangyu Wang, Zaihong Hou, Laian Qin, Xu Jing, Yi Wu

**Affiliations:** 1 Key Laboratory of Atmospheric Optics, Anhui Institute of Optics and Fine Mechanics, Hefei Institutes of Physical Science, Chinese Academy of Sciences, Hefei 230031, China; 2 Science Island Branch of Graduate School, University of Science and Technology of China, Hefei 230026, China; 3 Advanced Laser Technology Laboratory of Anhui Province, Hefei 230037, China

**Keywords:** imaging systems, wavefront reconstruction, lens array, Zernike polynomial, beam quality, large aperture measurement

## Abstract

In order to solve the problem of atmospheric influence on the far-field measurement of the quality of a laser beam, we proposed a direct wavefront measurement system based on the Hartmann detection principle, which can measure large apertures and high-power laser beams. The measuring system was composed of a lens array and a detector. The wavefront detection of a large aperture laser beam could be realized by controlling the distance between the lenses and the size of the lens. The influence of different duty cycle factors on the accuracy of the wavefront reconstruction under the same arrangement and different arrangement conditions was simulated and analyzed. The simulation results showed that when the sub-lenses of the system were not in close contact, the reconstruction accuracy of the duty factor of 0.8 was close to that of the case of the duty factor of 1. Within a certain detection range, the hexagonal arrangement of 19 lenses and the arrangement of 8 × 8 lens arrays had a high wavefront restoration accuracy; both were lower than 0.10 λ. The system proposed in this paper was suitable for measuring a large aperture laser beam, providing a new idea for measuring and analyzing the quality of large aperture laser beams. It also has an important significance for improving the measurement accuracy of the beam quality.

## 1. Introduction

In recent years, with its rapid development, laser technology has been more widely used in industry, medicine, national defense and other fields [1], so all walks of life have higher and higher requirements for the quality of laser beams. The premise of improving the beam quality is better laser measurement technology. At present, the main method is to measure the spatial and temporal distribution of the laser intensity in the far-field to obtain the relevant laser beam parameters [2,3,4]. Researchers have proposed a number of measurement methods and means for the measurement of the laser intensity, including the photosensitive method, scanning method, ablation method, camera imaging method and array detector method [5,6,7,8,9,10]. As a direct measurement method of the temporal and spatial distribution of the laser intensity, the array detector method is widely used in the field of laser measurement because of its high signal-to-noise ratio and good real-time performance. However, when the laser is transmitted in the atmosphere, it may be affected by turbulence disturbance, molecular absorption and other aspects [11], which makes it difficult for the measurement system to accurately obtain the beam parameters at the laser system outlet [12]. Therefore, it is of great significance to find a method that can greatly reduce or avoid the influence of the atmosphere to improve the measurement accuracy and carry out related research.

Aiming at atmospheric disturbance in the laser measurement system, if the beam parameters can be measured directly at the exit of the system, the influence from the atmosphere can be avoided. A Hartmann–Shack wavefront sensor can measure not only the phase distribution but also the intensity distribution of the light field [13]. Rao et al. [14] used the slope structure correlation coefficient and slope normalization coefficient measured by a Hartman wavefront sensor to measure the atmospheric turbulence parameters. Kovadlo [15] studied the features of the formation of the phase distortions of a light wave in order to determine the effect of turbulent layers in the atmosphere on the shift of these distortions within the images of sensor sub-apertures. Goodwin [16] improved the detection of atmospheric turbulence with SLODAR (slope detection and ranging). Nicholas [17] proposed a non-propagation method for turbulence simulation imaging combined with the Zernike coefficient, and applied it to ground-to-ground imaging. Han et al. [18] proposed an iterative extrapolation method, which could make each sub-spot find its corresponding sub-lens when the light beam passing through each sub-lens of the system was not in the corresponding sub-lens region to achieve the purpose of expanding the dynamic range of the Shack–Hartmann wavefront sensor. However, for the wavefront distribution of large aperture spots, especially the wavefront distribution of a high-power laser, the Hartmann–Shack wavefront detection method is not applicable due to the limitation of the aperture size and the detection threshold of the detection components [19].

In this paper, we propose a direct wavefront measurement system based on the Hartmann detection principle, which could measure a large aperture and a high-power laser beam. The measuring system was composed of a lens array and a detector. The wavefront detection of the laser spot was realized by controlling the distance between the lenses, the lens size and the focal length of the lens. When the distorted wavefront passed through the lens array of the system, the focus position measured on the focal plane of each sub-lens was offset to a certain extent, relative to the ideal plane wave. According to the offset, the average slope of the wavefront in each sub-lens region could be obtained and then the phase distribution information of the distorted wavefront could be obtained through a wavefront reconstruction algorithm [20,21]. According to the detected near-field phase, the point spread function representing the far-field characteristics could be calculated and then the beam quality could be evaluated and analyzed. The method proposed in this paper was suitable for measuring and analyzing a large aperture and a high-power laser system at the outlet, avoiding the influence of the atmosphere. The system structure was simple, the difficulty of implementation was low and the measurement accuracy was high.

## 2. System Measurement Principle and Evaluation Method

### 2.1. System Measurement Principle

It is feasible to use the lens array method to measure the wavefront distribution of a large aperture laser beam. This method is different from the traditional Hartmann wavefront sensor in that the lens elements are not connected. By adjusting the lens size and lens spacing, the near-field detection of a large aperture beam at the system exit is realized. When the incident light is an ideal plane wave, the focal plane of the lens array will be a group of evenly distributed focal points coincident with the center of each sub-lens. On the contrary, when the incident light is distorted, the focal points on the focal plane of the lens will no longer be uniformly arranged, but will have a certain offset position from the focal points of each sub-lens in an ideal situation [22,23]. According to the offset, the wavefront slope within each sub-lens region can be obtained and then the wavefront shape of the incident beam can be reconstructed through a wavefront reconstruction algorithm [20,21,24,25].

**Figure 1:**
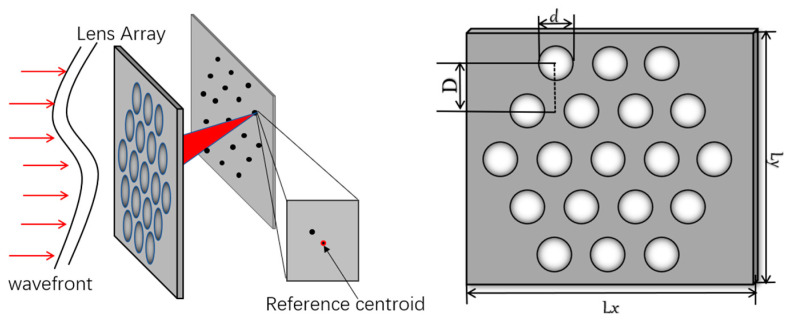
System structure diagram.

As shown in Figure 1, the incident laser irradiates on the lens array. If the incident laser is strictly a plane wave, the spot array formed on the detection surface is in the same position as the center of the lens array. When the wavefront of the laser beam is distorted, the focus of the beam in the focal plane of each sub-lens in the system lens array will not be in the center of the lens, but will have a certain offset Δ relative to the plane wave. The displacement of the *i*th lens in the x and y directions is defined as Δ*x_i_* and Δ*y_i_*, respectively, and the distance between the lens array and the detection plane is set as *f*. The slope of the wave surface on the *i*th lens in the x and y directions is then as follows:

(1) $$\begin{array}{l} g_{x_{i}}=\frac{\Delta x_{i}}{f}\\ g_{y_{i}}=\frac{\Delta y_{i}}{f} \end{array}$$

Therefore, *g_xi_* and *g_yi_* can be obtained by measuring Δ*x_i_* and Δ*y_i_*. After calculating *g_xi_* and *g_yi_*, the corresponding algorithm can be used to reconstruct the wavefront phase of the incident beam [22].

### 2.2. Wavefront Reconstruction Algorithm

The wavefront phase distribution can be expressed by Zernike polynomials:

(2) $$\Phi (x,y)=\sum_{k=1}^{l}a_{k}z_{k}(x,y)$$

where *l* is the number of modes, $a_{k}$ is the coefficient of the *k*th Zernike polynomial and $z_{k}$ is the *k*th Zernike polynomial.

The standard orthogonal set of Zernike polynomials can be constructed by the summation process. This is achieved by creating a single Zernike polynomial and then dividing it by its norm. The terms of the Zernike polynomial can be defined as follows:

(3) $$\begin{array}{l} Z_{evenj}=\sqrt{n+1}R_{n}^{m}(r)\sqrt{2}\cos(m\theta )m\neq 0\\ Z_{oddj}=\sqrt{n+1}R_{n}^{m}(r)\sqrt{2}\sin(m\theta )m\neq 0\\ Z_{j}=\sqrt{n+1}R_{n}^{0}(r)m=0 \end{array}$$

where

(4) $$R_{n}^{m}(r)=\sum_{s=0}^{(n-m)/2}\frac{{\left(-1\right)}^{s}(n-s)!}{s![(n+m)/2-s]![(n-m)/2-s]}r^{\left(n-2s\right)}$$

The wavefront slope of the sub-aperture can be expressed as

(5) $$G_{x}(x,y)=\sum_{k=1}^{l}a_{k}\frac{\partial z_{k}(x,y)}{\partial x}$$

(6) $$G_{y}(x,y)=\sum_{k=1}^{l}a_{k}\frac{\partial z_{k}(x,y)}{\partial y}$$

where *G_x_* and *G_y_* are the wavefront slopes in the *x* and *y* directions, respectively.

As the system detects only the average slope in the sub-aperture, *S_i_* is defined as the normalized area of the *i*th sub-aperture. Thus, the above formula can be modified as

(7) $$G_{x}{\left(x,y\right)}_{i}=\sum_{k=1}^{l}a_{k}\frac{\iint_{s_{i}}\frac{\partial Z_{k}{\left(x,y\right)}_{i}}{\partial x}dxdy}{S_{i}}=\sum_{k=1}^{l}a_{k}Z_{{}_{xk}}{}_{\left(i\right)}$$

(8) $$G_{y}{\left(x,y\right)}_{i}=\sum_{k=1}^{l}a_{k}\frac{\iint_{s_{i}}\frac{\partial Z_{k}{\left(x,y\right)}_{i}}{\partial y}dxdy}{S_{i}}=\sum_{k=1}^{l}a_{k}Z_{{}_{yk}}{}_{\left(i\right)}$$

Formula (8) is expressed by the matrix as

(9) $$\left[\begin{array}{l} G_{x}{}_{\left(1\right)}\\ G_{y}{}_{\left(1\right)}\\ G_{x}{}_{\left(2\right)}\\ G_{y}{}_{\left(2\right)}\\ \ldots \\ G_{x}{}_{\left(m\right)}\\ G_{y}{}_{\left(m\right)} \end{array}\right]=\left[\begin{array}{l} Z_{x1}{}_{\left(1\right)}Z_{x2}{}_{\left(1\right)}\ldots Z_{xN}{}_{\left(1\right)}\\ Z_{y1}{}_{\left(1\right)}Z_{y2}{}_{\left(1\right)}\ldots Z_{yN}{}_{\left(1\right)}\\ Z_{x1}{}_{\left(2\right)}Z_{x2}{}_{\left(2\right)}\ldots Z_{xN}{}_{\left(2\right)}\\ Z_{y1}{}_{\left(2\right)}Z_{y2}{}_{\left(2\right)}\ldots Z_{yN}{}_{\left(2\right)}\\ \ldots \;\ldots \;\ldots \;\ldots \\ Z_{x1}{}_{\left(m\right)}Z_{x2}{}_{\left(m\right)}\ldots Z_{xN}{}_{\left(m\right)}\\ Z_{y1}{}_{\left(m\right)}Z_{y2}{}_{\left(m\right)}\ldots Z_{yN}{}_{\left(m\right)} \end{array}\right]\cdot \left[\begin{array}{l} a_{1}\\ a_{2}\\ \ldots \\ a_{N} \end{array}\right]$$

where *m* is the total number of lens elements and *N* is the number of Zernike polynomials, which can be simplified as

(10) G=Z • A

Here, **G** is the calculated wavefront slope matrix, **Z** is the 2 m × *N* reconstruction matrix and **A** is the Zernike polynomial coefficient matrix that we needed to calculate. The specific solution of matrix **A** can be achieved by a matrix operation. In general, twice the total number of lenses is larger than the number of Zernike terms, so the singular value decomposition method can be used to calculate the generalized inverse matrix **Z^+^** of **Z**. The matrix **A** is given by

(11) $$A=Z^{+}•\;G$$

After the coefficient matrix is calculated, the wavefront can be reconstructed by substituting it back into Formula (2).

### 2.3. Evaluation Method

Mode coupling and aliasing should be considered when using Zernike polynomials for the wavefront reconstruction of a distorted wavefront. Mode coupling is caused by the fact that the number of Zernike terms selected during the wavefront reconstruction is smaller than the number of mode terms of the actual distorted wavefront. The reason for mode aliasing is that high-order aberrations and low-order aberrations cannot be effectively distinguished within the sub-lens range of the system [26]. Dai and Zhang et al. [27,28] also discussed the relationship between aliasing and cross-coupling and the number of Zernike terms used in wavefront reconstructions. By selecting different wavefront reconstruction orders, corresponding wavefront reconstruction accuracy and stability curves are obtained and then the final wavefront reconstruction orders are determined by a comparison. Furthermore, a turbulent wavefront is mainly composed of low-order Zernike polynomials and the proportion of high-order Zernike polynomials is very small. Therefore, we selected the first 14 Zernike polynomials without a piston for the wavefront reconstruction [21].

In this paper, we used the root mean square error (RMSE) as the standard to measure the accuracy of the wavefront reconstruction. The RMSE represents the root mean square value of the wavefront residual; the smaller the value, the higher the accuracy of the restoration. The phase information of a residual wavefront is obtained by subtracting the reconstruction wavefront and the incident wavefront, and then the RMS is calculated. The calculation formula is:

(12) $$\varepsilon_{RMSE}=\sqrt{({\sum_{u}\left(\phi (u)-\phi_{o}(u)\right)}^{2})/N}$$

where *ϕ_o_*(*u*) is the original wavefront, *ϕ*(*u*) is the recovery wavefront and *N* is the total number of sampling points.

## 3. Results and Discussion

### 3.1. System Input Parameters

It has been explained above that a turbulence wavefront aberration is mainly composed of low-order Zernike polynomials and the proportion of high-order Zernike polynomials is very small. Therefore, the first 14 terms of the Zernike polynomials without a piston were selected. Based on this, we analyzed the two situations of more and fewer lenses, and proposed three different lens array arrangements: an 8 × 8 lens array arrangement; a 5 × 5 lens array arrangement; and a hexagonal arrangement of 19 lenses. The 8 × 8 lens array arrangement and 5 × 5 lens array arrangement were used to analyze the reconstruction accuracy of a distorted wavefront with a high spatial resolution system structure and a low spatial resolution system structure, respectively. The 5 × 5 lens array arrangement and 19-element hexagon arrangement were used to analyze the reconstruction accuracy of a distorted wavefront with a different lens arrangement under the condition of a similar spatial resolution.

Before the simulation and comparison of the wavefront restoration ability of the three different arrangement modes, it was necessary to first determine the lens size, lens focal length, duty cycle factor and other parameters of the system. As the Zernike mode method used in wavefront reconstructions is based on centroid detection, the centroid detection accuracy is particularly important. To improve the measurement accuracy of the centroid of the sub-spot, it is particularly important to ensure that the size of the sub-spot on the detection surface should occupy more pixels and the minimum pixel size of the sub-spot should be 4 pixels × 4 pixels [29]. In order to ensure the measurement accuracy, this system set the pixel range covered by the sub-spot to 10 pixels × 10 pixels. According to 2.44λ/d, the diffraction limit angle of a beam passing through a single lens can be known, and the number of pixels occupied by the light spot can be determined according to the diffraction limit angle:

(13) $$p=2.44\frac{\lambda }{d}\cdot \frac{f}{P}$$

**Figure 2:**
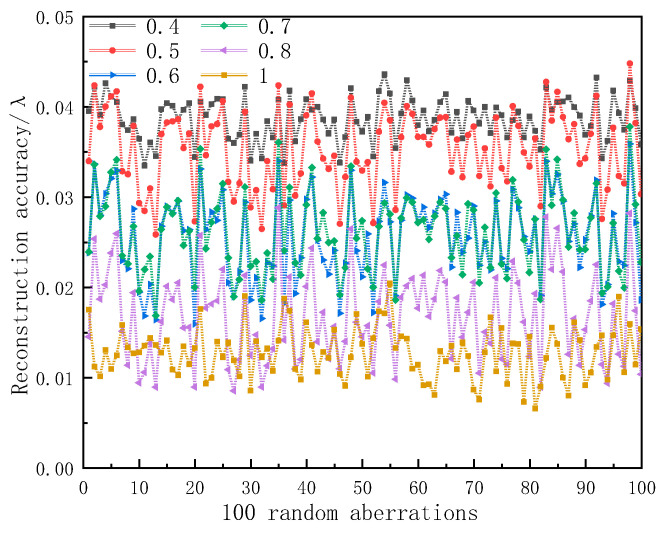
Wavefront reconstruction accuracy of 8 × 8 lens array with different duty cycles.

Here, *P* = 5.5 μm is the pixel size and *λ* = 1064 nm is the wavelength. If *p* ≥ 10, then *f* ≥ 22 d. The lens size we chose was 25.4 mm and then the focal length of the lens could be determined. By adding a diaphragm in front of the lens, the accuracy of the wavefront reconstruction was compared under different duty cycle factors. Taking the arrangement of the 8 × 8 lens array as an example, under the conditions of different duty cycle factors, the reconstruction accuracy results for the same group of random wavefront aberrations are shown in Figure 2. The random aberration here was based on the systematic aberration of a solid-state laser.

It can be seen from Figure 2 that under the same arrangement, the larger the fill factor of the lens, the higher the accuracy of the system in restoring the wavefront. When the duty cycle of the lens array was 0.8, the accuracy of the reconstructed wavefront of the system was close to that when the duty cycle of the lens array was 1. One of the purposes of this system design was to use a combination of smaller lenses to detect as large a distorted wavefront as possible, so we chose the duty factor of the system lens array to be 0.8.

**Figure 3:**
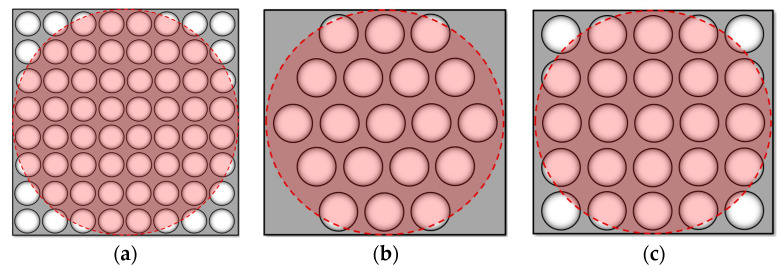
System sub-aperture layout: (**a**) 8 × 8 lens array; (**b**) hexagonal arrangement of 19 lenses; (**c**) 5 × 5 lens array.

As the lens size we chose was 25.4 mm, when the duty factor was 0.8, the effective size of the lens was 20 mm. Thus, we set the focal length of the lens to 500 mm accordingly. The overall input parameters of the system are shown in Table 1 and the lens arrangement is shown in Figure 3. For the 8 × 8 case, the lens size was reduced accordingly; the red area in the figure was the effective detection range of the incident beam.

### 3.2. Single Aberration Analysis

**Figure 4:**
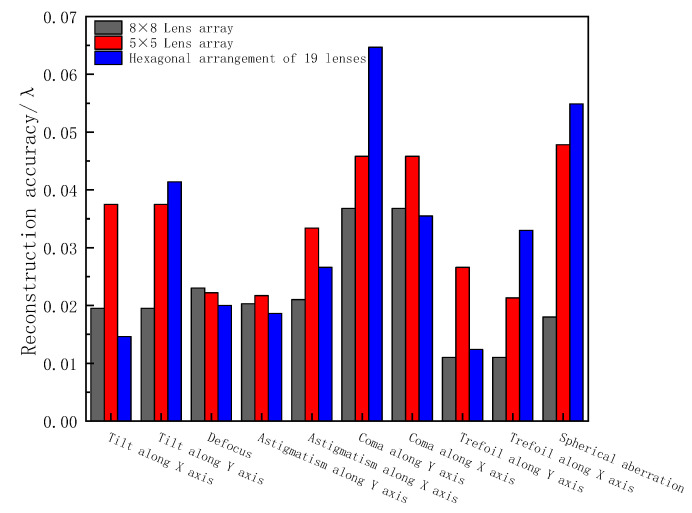
Reconstruction accuracy of a single aberration by three arrangement methods.

First, the above three arrangements were used to analyze a low-order single aberration. The simulation results are shown in Figure 4.

From the reconstruction results of each single aberration, the reconstruction accuracy of the 8 × 8 lens array for each order of single aberrations was better than the hexagonal arrangement of 19 lenses and the 5 × 5 lens array arrangement. For different types of aberration wavefronts, different arrangements will also bring about different reconstruction accuracy characteristics. Both the 8 × 8 array arrangement and the 5 × 5 array arrangement were center-symmetrical structures, so the reconstruction accuracy in the x-direction and y-direction for the aberrations in the two fixed directions of tilt and coma was consistent. However, due to its structural characteristics, the hexagonal arrangement had a better reconstruction accuracy of aberrations in the x-direction than in the y-direction for the aberrations in a fixed direction.

### 3.3. Combined Aberration Analysis

**Figure 5:**
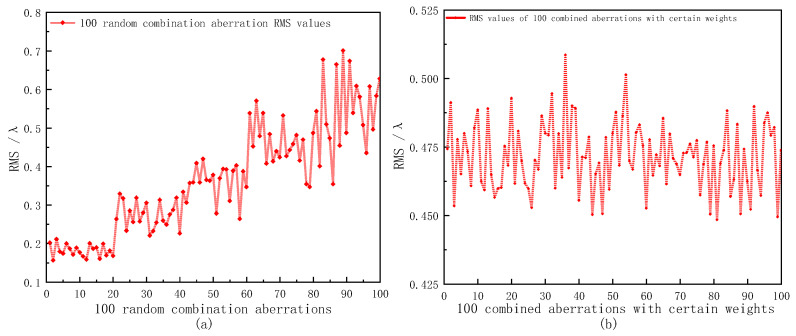
RMS values of 100 combined aberrations: (**a**) random combined aberrations; (**b**) combined aberrations with certain weights.

We selected the first 14 Zernike polynomials without the piston term, which basically represented the low-frequency components of a wavefront phase. In order to reflect the recovery ability of the system to the combined aberration, the analysis was mainly carried out from two aspects. One was to generate 100 groups of random aberrations with gradually increasing wavefront RMS values by substituting random coefficients within a certain range into Formula (2). The other was to analyze the wavefront aberration of a specific laser system. Zhang et al. [30] analyzed the wavefront aberration of an output beam of a laser diode-pumped Nd:YAG solid laser, mainly showing defocusing, low-order astigmatism and a spherical aberration. The corresponding coefficient reference values are shown in Table 2. According to the Zernike coefficient values of each order, random values could be selected at a small interval around it to generate a combined aberration with a certain weight aberration. The added random quantities ranged from [−0.1, 0.1]. Figure 5 shows the RMS curves of the two generated combined aberrations.

**Figure 6:**
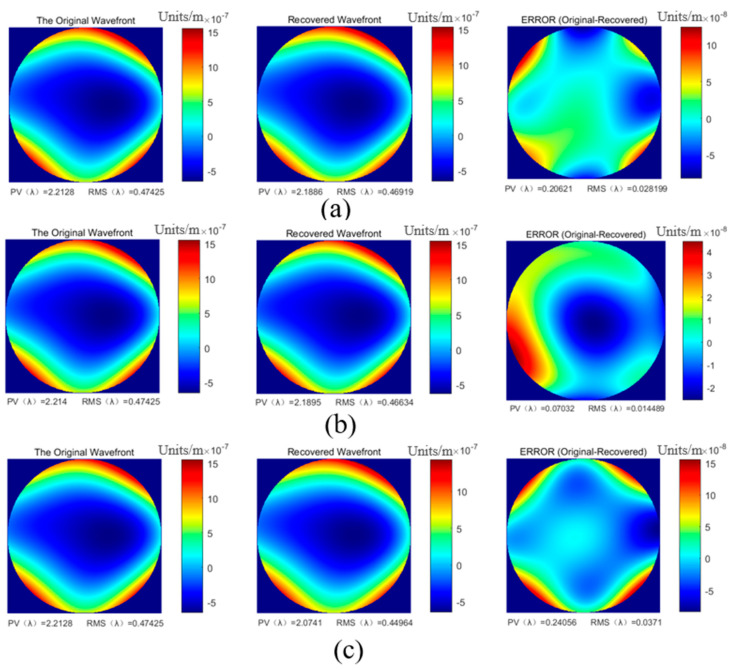
Simulation example of wavefront reconstruction: (**a**) hexagonal arrangement of 19 lenses; (**b**) 8 × 8 lens array; (**c**) 5 × 5 lens array.

The formed wavefront was processed and calculated by the lens array system to obtain the reconstructed wavefront and then the accuracy of the wavefront reconstruction was calculated. Figure 6 shows one of the simulation examples. The above three arrangements were used to reconstruct the same aberration. The wavefront PV (peak-to-valley) value of the measured aberration was 2.21 λ and the RMS value was 0.474 λ.

**Figure 7:**
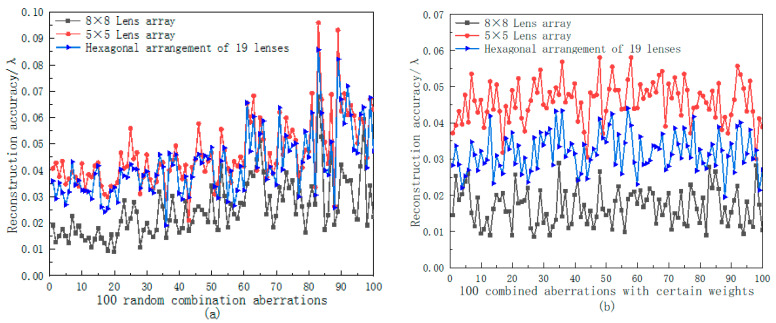
Reconstruction accuracy of different combination aberrations by three arrangement methods: (**a**) random combined aberrations; (**b**) combined aberrations with certain weights.

The reconstruction accuracy of the two combined aberrations and the statistical values of the reconstructed data of the three arrangement methods are shown in Figure 7, Table 3 and Table 4, respectively.

Combining Figure 7, Table 3 and Table 4, it can be seen that for the random combined aberrations, the 8 × 8 lens array had the best reconstruction accuracy and the most stable reconstruction results. The average reconstruction accuracy for 100 groups of random combined aberrations and combined aberrations with a certain weight was 0.025 λ and 0.0167 λ, respectively. As the arrangement of the 5 × 5 lens array was the same as that of the 8 × 8 lens array, the trend of the reconstruction accuracy of the two arrangements for the same aberration was basically the same. However, it was limited by the small number of lenses, so its average reconstruction accuracy for the two combined aberrations was 0.046 λ and 0.0459 λ, respectively. Although the hexagonal arrangement of 19 lenses had fewer lenses, its arrangement had a greater space utilization rate, so the reconstruction accuracy of the aberration was better than that of the 5 × 5 array arrangement. The average reconstruction accuracies of the two combined aberrations were 0.042 λ and 0.032 λ, respectively.

## 4. Conclusions

In this paper, we proposed a wavefront direct measurement system based on the Hartmann detection principle that could measure large aperture laser beams. A system structure model of different lens arrangements was established and the influence of different duty factors on the accuracy of wavefront reconstructions under the same arrangement was analyzed. The wavefront reconstruction accuracy of each single aberration and random combined aberration was compared with the three arrangements of an 8 × 8 lens array, a 5 × 5 lens array and a hexagonal arrangement of 19 lenses. The results showed that when the sub-lenses of the system were not in close contact, the reconstruction accuracy was close to 1 when the duty factor was 0.8. In addition, whether it was a random combined aberration or a combined aberration with a certain weight, the reconstruction accuracy of the 8 × 8 lens array was the highest and the average reconstruction accuracies were 0.025 λ and 0.017 λ, respectively. The average reconstruction accuracy of the random combined aberrations of the 5 × 5 lens array arrangement and the hexagonal arrangement of 19 lenses were 0.046 λ and 0.042 λ, respectively. For the combined aberration with a certain weight, the reconstruction accuracy of the hexagonal arrangement of 19 lenses was significantly higher than that of the 5 × 5 lens array arrangement. The above results showed that a system structure with a high spatial resolution could reconstruct a distorted wavefront more accurately; when the number of lenses was small, improving the space utilization of the detection system could also improve the wavefront reconstruction accuracy. In summary, the new wavefront measurement system proposed in this paper had a high reconstruction accuracy of an incident wavefront, providing a new idea for solving the problem of the beam quality measurement of large aperture laser beams.

## Figures and Tables

**Table 1:** System input parameters.

Parameter	Description
Beam aperture	125 mm
Beam wavelength	1064 nm
Lens size	20 mm
Lens spacing	5 mm
Duty factor	0.8
Lens focal length	500 mm

**Table 2:** The definition and weight value of the first 10 Zernike aberration orders.

Order	Aberration	Zernike Coefficient
Z1	X tilt	−2.372
Z2	Y tilt	−0.433
Z3	Defocus	0.464
Z4	0/90°Astigmatism	−0.053
Z5	45/135°Astigmatism	−0.183
Z6	X coma	0.062
Z7	Y coma	0.035
Z8	High-order astigmatism	−0.014
Z9	High-order astigmatism	−0.063
Z10	Spherical aberration	0.059

**Table 3:** Reconstruction accuracy statistics of 100 random combination aberrations by three arrangement methods.

	Quantity	Mean	Standard Deviation
8 × 8 Lens array	100	0.02529	0.01114
5 × 5 Lens array	100	0.04611	0.01286
Hexagonal arrangement of 19 lenses	100	0.04224	0.01265

**Table 4:** Reconstruction accuracy statistics of 100 combination aberrations with certain weights by three arrangement methods.

	Quantity	Mean	Standard Deviation
8 × 8 Lens array	100	0.01668	0.0051
5 × 5 Lens array	100	0.04588	0.00572
Hexagonal arrangement of 19 lenses	100	0.03185	0.00534

## Data Availability

Data are available upon request.

## References

[1] Li G.P., Chen C., Li D., Wu L., Zhang B., Yu D.J., Yin W.H. (2020). Study on parameters measurement technology of high energy and high power laser. J. Appl. Opt..

[2] Pang M., Zhou S., Wu J. (2013). Research on scattering sampling and attenuating in measurement of laser intensity distribution. Infrared Laser Eng..

[3] Guan W.L., Tan F.F., Hou Z.H., Luo J., Qin L.A., He F., Zhang S.L. (2022). Wide Angle Array Detection Technology for High Power Density Laser. Acta Opt. Sin..

[4] Scharmer G.B., Van W.T.I.M. (2010). S-DIMM+ height characterization of day-time seeing using solar granulation. Astron. Astrophys..

[5] Du X.W. (1997). Factors for Evaluating Beam Quality of a Real High Power Laser on the Target Surface in Far Field. Chin. J. Lasers.

[6] Gao W., Wang Y.P., Li B. (2003). Study on characterization and diagnosis of high-power laser beam quality. Infrared Laser Eng..

[7] Feng G.Y., Zhou S.H. (2009). Discussion of Comprehensive Evaluation on Laser Beam Quality. Chin. J. Lasers.

[8] Feng G.B., Wang Z.B., Feng G. (2013). Detector Array for Measuring Temporal-Spatial Distribution of High-Repetition-Rate Pulsed Laser Beam Profile. Chin. J. Lasers.

[9] Feng G.B., Yang P.L., Wang Q.S. (2013). Measuring technology for far-field beam profile of high power laser. High Power Laser Part. Beams.

[10] Gui G., Brooks N.J., Wang B., Kapteyn H.C., Murnane M.M., Liao C.T. (2022). Single-Frame Characterization of Ultrafast Pulses with Spatiotemporal Orbital Angular Momentum. ACS Photonics.

[11] Ye Z.M. (2017). Error Analysis of Laser Far Field Characteristics Measurement Based on Detector Array. Electro-Opt. Technol. Appl..

[12] Chen Y., Wang S., Xu Y., Dong Y. Simulation and Analysis of Turbulent Optical Wavefront Based on Zernike Polynomials. Proceedings of the 2013 IEEE International Conference on Green Computing and Communications and IEEE Internet of Things and IEEE Cyber, Physical and Social Computing.

[13] Duan H.F., Yang Z.P., Wang S.Q., Zhang Y.D. (2002). Model Wavefront Reconstruction of Shack-Hartmann Sensor on Arbitrary Area and Wavefront Expression by Zernike Polynomials. Chin. J. Lasers.

[14] Rao C.H., Jiang W.H., Ling N. (2000). Atmosperic Parameters Measurements for Non-Kolmogorov Turbulence with Hartmann-Shack Wavefront Sensor. Acta Opt. Sin..

[15] Kovadlo P.G., Shikhovtsev A.Y., Kopylov E.A., Kiselev A.V., Russkikh I.V. (2021). Study of the Optical Atmospheric Distortions using Wavefront Sensor Data. Russ. Phys. J..

[16] Goodwin M., Jenkins C., Lambert A. (2007). Improved detection of atmospheric turbulence with SLODAR. Opt. Express.

[17] Chimitt N., Chan S.H. (2020). Simulating anisoplanatic turbulence by sampling intermodal and spatially correlated Zernike coefficients. Opt. Eng..

[18] Han Y.N., Hu X.Q., Dong B. (2020). Iterative Extrapolation Method to Expand Dynamic Range of Shack-Hartmann Wavefront Sensors. Acta Opt. Sin..

[19] Wu W.W., Sun H.Y., Song F.H. (2009). A New Wavefront Reconstruction Method Based on Simulate H-S Sensor. J. Equip. Acad..

[20] Dai F.Z., Zheng Y.Z., Bu Y., Wang X.Z. (2016). Zernike polynomials as a basis for modal fitting in lateral shearing interferometry: A discrete domain matrix transformation method. Appl. Opt..

[21] Noll R.J. (1976). Zernike polynomials and atmospheric turbulence. JOSA.

[22] Liu L.Q., Zhang Q.Q., Jing F., Peng Z.T., Zhu Q.H. (2001). Two phase reconstruct algorithms of high power laser beam. High Power Laser Part. Beams.

[23] Hu S.J., Xu B., Hou J. (2002). Application of Hartmann-Shack Wavefront Sensor to Measurement of Beam Quality Factor M~2. Opto-Electron. Eng..

[24] Mahajan V.N. (1995). Zernike polynomials and optical aberrations. Appl. Opt..

[25] Bernd S., Maik L., Klaus M. (2006). Propagation of laser beams from Hartmann-Shack measurements. Photon. North.

[26] Herrmann J. (1981). Corss coupling and aliasing in modal wavefront estimation. Opt. Soc. Am..

[27] Dai G.M. (1996). Modal wavefront reconstruction with Zernike Polynomials and Karhunen-Loeve function. Opt. Soc. Am..

[28] Zhang Q., Jiang W.H., Xu B. (1998). Reconstruction of Turbulent Optical Wavefront Realized by Zernike Polynomial. Opto-Electron. Eng..

[29] Liu M.S., Wang X.M., Jing W.B., Wang B. (2013). Design of Parameters of Shack-Hartmann Wave-front Sensor for Laser-Beam Quality Meersurement. Acta Opt. Sin..

[30] Zhang X., Su L.K., Cai Q. (2010). Analysis of Thermal Effect and Experimental Test of Beam Wavefront Aberration in All Solid-State Nd:YAG Laser. Acta Opt. Sin..

